# Mitochondria-Targeting Chemodynamic Therapy Nanodrugs for Cancer Treatment

**DOI:** 10.3389/fphar.2022.847048

**Published:** 2022-01-10

**Authors:** Qiaohui Chen, Niansheng Li, Xiaoyuan Wang, Yuqi Yang, Yuting Xiang, Xingyu Long, Jinping Zhang, Jia Huang, Li Chen, Qiong Huang

**Affiliations:** ^1^ Xiangya School of Pharmaceutical Sciences, Central South University, Changsha, China; ^2^ Hunan Provincial Key Laboratory of Cardiovascular Research, Xiangya School of Pharmaceutical Sciences, Central South University, Changsha, China; ^3^ Department of Pharmacy, Xiangya Hospital, Central South University, Changsha, China; ^4^ National Clinical Research Center for Geriatric Disorders, Xiangya Hospital, Central South University, Changsha, China

**Keywords:** chemodynamic therapy, mitochondria-targeting, nanomaterials, cancer therapy, reactive oxygen species

## Abstract

Mitochondria, as one of the most critical subcellular organelles of cancer cells, are very vulnerable and often on the verge of oxidative stress. The classic chemodynamic therapy (CDT) directly employs endogenous chemical energy to trigger reactive oxygen species (ROS) burst and destroy tumor cells. However, the effectiveness of CDT is restricted by the limited diffusion distance and short half-life of ROS. From this perspective, the treatment method (mitochondria-targeting chemodynamic therapy nanodrugs, M-CDT nanodrugs) that can generate high levels of ROS at the mitochondrial site is extremely efficient and promising for cancer treatment. Currently, many emerging M-CDT nanodrugs have been demonstrated excellent spatial specificity and anti-cancer efficacy. In this minireview, we review various proof-of-concept researches based on different M-CDT nanodrugs designs to overcome the limits of the efficacy of CDT, mainly divided into four strategies: supplying H_2_O_2_, non-H_2_O_2_ dependent CDT, eliminating GSH and enhancing by hyperthermia therapy (HT). These well-designed M-CDT nanodrugs greatly increase the efficacy of CDT. Finally, the progress and potential of M-CDT nanodrugs are discussed, as well as their limitations and opportunities.

## Introduction

Many new therapeutic targets and emerging therapies have been developed to find the Achilles heel of cancer cells (de Lázaro and Mooney, 2021). However, most therapy methods cannot completely kill malignant tumors or prevent the formation and recurrence of metastatic tumors (Chen et al., 2020). Mitochondria, as one of the most critical organelles in cancer cells, play a vital role in the energy supply, calcium homeostasis regulation, and signal transduction of cancer cells (Jiang et al., 2021). Mitochondria are also the key regulators of the apoptosis pathway. Damaged mitochondria release pro-apoptotic proteins (like cytochrome c) from the mitochondrial membrane space to the cytoplasm to trigger apoptosis in a typical apoptotic pathway (Bock and Tait, 2020). Moreover, the mitochondria of cancer cells usually undergo metabolic reprogramming for rapid proliferation and invasion, that is, they are more inclined to glycolysis than oxidative phosphorylation to produce ATP (Martínez-Reyes and Chandel, 2021). This process also produces a higher level of reactive oxygen species (ROS), which makes the mitochondria themselves on the verge of oxidative stress (Hayes et al., 2020). ROS has strong oxidative activity and can cause severe oxidative damage (Qin et al., 2021). Therefore, it is an extremely efficient strategy to promote mitochondrial damage to kill cancer cells by increasing the level of ROS in the mitochondrial region (Guo et al., 2021).

**FIGURE 1:**
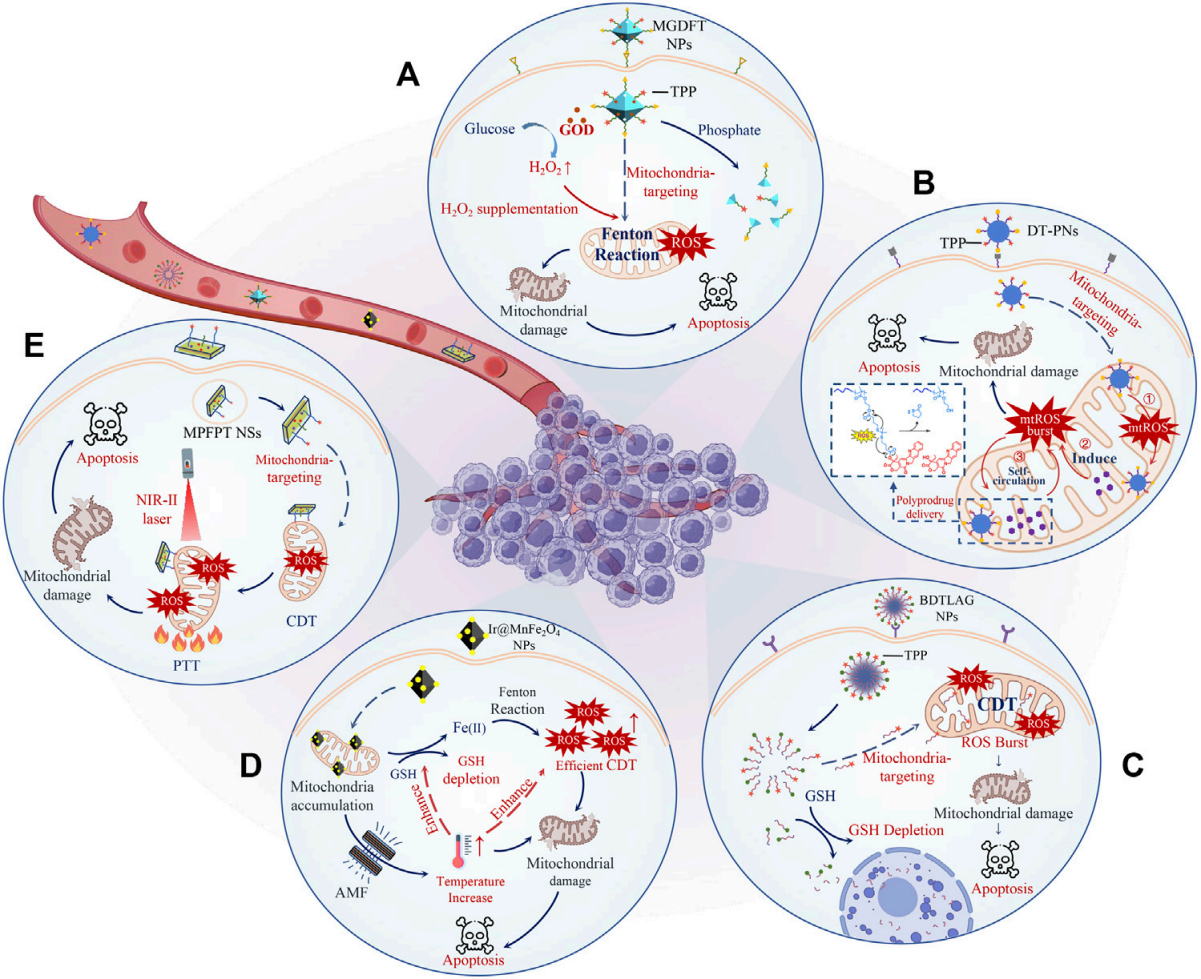
Schematic illustration of representative M-CDT nanodrugs. The therapeutic efficacy of M-CDT nanodrugs is further increased by H_2_O_2_ supplementation **(A)**, non-H_2_O_2_ dependent CDT **(B)**, GSH depletion **(C)**, and combination with MHT **(D)** or PTT **(E)**. To be specific, MGDFT NPs catalyzed the decomposition of glucose into H_2_O_2_ and gluconic acid through loaded GOD, significantly increasing the level of H_2_O_2_ **(A)**. DT-PNs delivered non-H_2_O_2_ CDT agents to mitochondria through a poly-prodrug delivery strategy, triggering a ROS burst *in situ* and efficiently killing tumor cells **(B)**. BDTLAG NPs rapidly consumed GSH and released CDT agents through the reaction of disulfide bonds with GSH, which significantly improved the tumor killing effect of ROS **(C)**. Ir@MnFe_2_O_4_ NPs generated high temperatures locally in the mitochondria under the AMF, greatly promoting the rate of Fenton reaction and thus producing a high level of ROS to induce mitochondria damage **(D)**. MPFPT NSs with high photothermal conversion efficiency efficiently accelerated the rate of the Fenton reaction by heating mitochondria, indicating the superiority of PTT-CDT with mitochondria targeting **(E)**.

ROS-based cancer therapy such as chemodynamic therapies (CDT), photodynamic therapy (PDT), and sonodynamic therapy (SDT) have attracted substantial attention in recent years with the advantages of noninvasiveness, high tumor specificity, minimal toxicity, and low drug resistance (Wang et al., 2021a; Gao et al., 2020a). Among them, CDT has more unique advantages than other methods because it directly employs endogenous H_2_O_2_ in tumor site to generate toxic ROS through Fenton/Fenton-like reactions (Eq. 1) or other ways to kill cancer cells with no need for external energy input or O_2_ (Tian et al., 2021). However, ROS is a short-lived and efficient “killer” with a lifetime of less than 0.1 ms and a diffusion range of only 10–20 nm (Zhang et al., 2019). The initial location of ROS production has a significant impact on the therapeutic effect of CDT. CDT agents with targeting mitochondria can produce a high level of ROS *in situ* on mitochondria to cause severely mitochondrial damage and further trigger cell death (Zeng et al., 2021). However, the transport of CDT agents to the mitochondria of cancer cells is a huge bottleneck. In addition, the effect of CDT is greatly limited by the high concentration of glutathione (GSH), the unsustainable supply of H_2_O_2_, and the low Fenton reaction rate at the tumor site (Wang et al., 2020). Fortunately, the mitochondrial membrane potential of cancer cells is much higher than that in normal cells (∼−220 mV vs. ∼ −140 mV) (Fantin et al., 2006; Bagkos et al., 2014), which means mitochondria targeting groups with positive electricity and lipophilicity can be preferentially enriched into mitochondria of cancer cells (Shen et al., 2020; Xie et al., 2021). Moreover, the rapid development of nanodrugs has brought new hope for an ideal CDT agent delivery system. Well-designed nanodrugs can greatly increase the penetration of CDT agents at tumor sites by the enhanced permeation and retention (EPR) effect or active targeting (Li et al., 2021a; Nadar et al., 2021). Currently, many emerging CDT nanodrugs with mitochondria-targeting (M-CDT nanodrugs) have recently been demonstrated excellent spatial specificity and anti-cancer efficacy. More importantly, the therapeutic efficacy of M-CDT nanodrugs is further increased by GSH depletion (Wang et al., 2021b), H_2_O_2_ supplementation (Sun et al., 2021), non-H_2_O_2_ dependent CDT (Zhang et al., 2019), and combination hyperthermia therapy. This minireview focuses on the latest progress of M-CDT nanodrugs because of the significance of M-CDT nanodrugs and many advances in this research field. We divide these M-CDT nanodrugs into two parts to comprehensively summarize: Firstly, self-enhanced M-CDT nanodrugs with H_2_O_2_ supplementation, non-H_2_O_2_ dependent CDT, and GSH depletion; Secondly, HT enhanced M-CDT nanodrugs (Figure 1; Table 1). Finally, we propose the future development direction and challenges of M-CDT nanodrugs. This review provides the most advanced treatment methods and valuable information for cancer treatment.

$$H_{2}O_{2}\mathrm{+F}e^{\mathrm{2+}}\to \mathrm{\cdot OH+O}H^{-}\mathrm{+F}e^{\mathrm{3+}}$$ (1)

**TABLE 1:** Nanodrugs for mitochondria-targeting chemodynamic therapy.

Category	Strategy	Nanomaterials	Tumor cell-targeting ligands	Mitochondria-targeting molecules	Size	Fenton agent	Adjuvant therapy
Self-enhanced M-CDT nanodrugs	H_2_O_2_ supplement	FG/T-Nanoprodrug (Sun et al., 2021)	—	TPP	142 nm	Ferrocene, LND	—
		MGDFT NPs (Peng et al., 2021)	FA	TPP	∼400 nm	Fe (II)	Chemotherapy (CT)
		FC-BBR/IND@GOD@HA NPs (Cheng et al., 2022)	HA	Berberine	156 nm	Ferrocene	CT
	non-H_2_O_2_ dependent CDT	DT-PNs(Zhang et al., 2019)	cRGD	TPP	∼55 nm	CPT	—
	GSH depletion	BDTLAG NPs (Wang et al., 2021b)	Biotin	TPP	∼140 nm	α-TOS, LND	CT
		CDTLG NPs (Wang et al., 2021c)	Glycyrrhetinic acid	TPP	∼147 nm	α-TOS, LND	CT
		TLDCAG NPs (Wang et al., 2021d)	—	TPP	∼185 nm	α-TOS, LND	CT
HT enhanced M-CDT nanodrugs	CDT + MHT	Ir@MnFe_2_O_4_ NPs (Shen et al., 2020)	—	Iridium (III) complexes	11.24 ± 1.11 nm	Fe (II)	MHT
	CDT + PTT	CuO@AuCu-TPP nanocarriers (Li et al., 2020)	—	TPP	255 nm	Cu (I)	PTT
		MPFPT NSs(Li et al., 2021c)	—	TPP	∼7.2 nm (thickness)	Fe (II)	PTT
		PTFHD nanoplatforms (Jin et al., 2020)	HA	TPP	120 nm	Fe (II)	PTT, CT
	CDT + PDT	TPP-PEG-Au–Ag cages (Wen et al., 2021)	—	TPP	∼50 nm	Ag (I)	PDT, PTT
		Fe_3_O_4_@Dex-TPP/PpIX/ss-mPEG NPs (Hou et al., 2019)	—	TPP	64.34 ± 2.63 nm	Fe (II)	PDT
		UCSRF(Hu et al., 2017)	—	Ru (II) complex	60–80 nm	Fe (II)	PDT

## Self-Enhanced M-CDT Nanodrugs

Cancer cells have a much higher level of H_2_O_2_ (up to 100 mM) than normal cells (20 nM), which is also the first prerequisite for CDT treatment (Tang et al., 2020). However, H_2_O_2_ cannot be continuously supplied, which greatly limits the effect of CDT (Xuan et al., 2020). In addition, cancer cells have high concentrations of GSH to balance the oxidative stress caused by ROS (Langford et al., 2018). The ROS can easily be eliminated by the GSH-Glutathione peroxidase system during CDT treatment. Currently, three strategies are developed to greatly improve the efficacy of M-CDT nanodrugs: supplying H_2_O_2_, non-H_2_O_2_ dependent CDT, and eliminating GSH in cancer cells.

H_2_O_2_ is still insufficient for continuous ·OH generation during Fenton reaction-based CDT. Therefore, M-CDT nanodrugs with H_2_O_2_-supplement are advantageous for maintaining continuous oxidative damage (Cheng et al., 2022). For example, Peng et al. (2021) designed phosphate-degradable nanoparticles based on metal-organic frameworks nanocarriers (MGDFT NPs) with glucose oxidase (GOD) and doxorubicin (DOX) for CDT of cancer. Mil-101 (Fe) was selected as the nanocarrier for DOX and GOD, as well as the iron source for the Fenton reaction. Then Mil-101 (Fe) was modified by folic acid (FA) and triphenylphosphonium (TPP) to get a sequential targeting efficiency to cancer cells and mitochondria in cancer cells, respectively. Furthermore, MIL-101(Fe) was phosphate-sensitive and easy to degrade in cancer cells with the high concentration of phosphate, and MGDFT NPs disintegrated quickly to release iron ions and GOD. GOD catalyzed efficiently the reaction of glucose and O_2_ to produce gluconic acid and H_2_O_2_, which supplied the Fenton reaction substrate and prolonged CDT treatment duration. The level of ROS produced by MGDFT NPs was 4.4 times that of the NPs without GOD in 4T1 cells. The growth of cancer cells was successfully inhibited by MGDFT NPs without obvious side effects. Similarly, Sun et al. (2021) developed M-CDT nanodrugs loaded with GOD and lonidamine (FG/T-Nanoprodrug) for the chemotherapy and CDT combination therapy. The FG/T-Nanoprodrug was prepared in two steps: First, a T-Prodrug containing polylysine, TPP, and lonidamine self-assembled to form nanoparticles; Secondly, GOD and ferrocene (a Fenton reaction catalyst) were linked to the surface of nanoparticles to prepare the FG/T-Nanoprodrug. The FG/T-Nanoprodrug entered the mitochondria of cancer cells mediated by TPP and then released lonidamine and GOD. Lonidamine, as a hexokinase inhibitor (the rate-limiting enzyme of glycolysis), reduced glucose utilization by inhibiting mitochondrial energy metabolism, which allowed more glucose to be converted by GOD into H_2_O_2_ in cancer cells. The FG/T-Nanoprodrug continuously produced abundant ROS to destroy mitochondria and release cytochrome c. Tumor tissues treated with the FG/T-Nanoprodrug had a 7-fold increase of ROS levels compared to the saline group after 7 days. The FG/T-Nanoprodrug demonstrated extremely strong tumor suppression and ablation in a tumor xenograft model.

Non-H_2_O_2_ dependent CDT completely avoids the bottleneck problem that H_2_O_2_ cannot be continuously supplied in cancer cells (Gao et al., 2020b). Some chemotherapy drugs can directly stimulate the generation of endogenous ROS by interacting with the mitochondrial respiratory chain complex, which is considered to have CDT effect as well (Wang et al., 2021c; Wang et al., 2021d). For example, camptothecin (CPT), as an atypical CDT agent, can not only inhibit DNA topoisomerase I but stimulate the production of endogenous mitochondrial ROS to hyperpolarize mitochondria (Sánchez-Alcázar et al., 2000; Sen et al., 2004). Recently, Zhang et al. (2019) developed ROS-responsive polyprodrug nanoreactors with dual-targeting properties (DT-PNs) for cancer therapy. DT-PNS was self-assembled with two polyprodrug amphiphiles connected with multi-CPTs through the ROS-responsive thioketal linkage: cRGD-PDMA-bPCPTSM and TPP-PDMA-bPCPTSM. The decorated cyclic Arg-Gly-Asp (cRGD) peptide and TPP targeted the overexpressed integrins on the surface of cancer cells and mitochondria, respectively. As a result, DT-PNs internalized by cancer cells precisely anchored the mitochondria and released the initial CPT in response to endogenous upregulated mitochondrial ROS, while the CPT released *in situ* further triggered mitochondrial ROS, and finally formed a CPT-ROS circulation to generate a ROS burst in mitochondria. The self-circulation of CPT release and ROS burst led to mitochondrial-dependent apoptosis and efficiently killed cancer cells.

Very recently, Wang et al. (2021b) constructed a sequential multi-stage targeting nanoparticles (BDTLAG NPs) to increase the effect of non-H_2_O_2_ dependent CDT by GSH depletion. The BDTLAG NPs consisted of three parts: two mitochondria targeting CDT agents (TPP-PEG_2K_-LND and TPP-PEG_2K_-TOS) and a GSH scavenging group (Bio-PEG_2K_-S-S-CPT). The TPP group mediated the mitochondria targeting of lonidamine (LND) and α-tocopheryl succinate (α-TOS), both of which interfered with the mitochondrial energy metabolism to stimulate endogenous ROS production. Importantly, the Bio-PEG_2K_-S-S-CPT part of BDTLAG NPs actively targeted cancer cells because the surface of the cancer cells expresses biotin (Bio) receptors. The disulfide bond in the BDTLAG NPs broke to release CPT, LND, and TOS by reacting with GSH in cancer cells. The effect of depletion GSH was measured by the released CPT. Bio-PEG_2K_-S-S-CPT effectively removed GSH in cancer cells, and the CPT released by Bio-PEG_2K_-S-S-CPT was 6.8 times higher than that by the group without GSH response in cancer cells. At the same time, LND and TOS entered the mitochondria and caused high concentrations of ROS *in situ* on mitochondria, which led to mitochondria damage to induce the death of cancer cells. Compared with the CPT group and the BDG NPs group (without mitochondria targeting), BDTLAG NPs had the best tumor suppression effect because of their mitochondria targeting and efficient GSH depletion.

## HT Enhanced M-CDT Nanodrugs

The slower reaction rate of CDT based on Fenton reaction is also a major factor limitation of the effectiveness of CDT (Cao et al., 2021). The high temperature from hyperthermia therapy (HT) can improve the rate of the Fenton/Fenton-like reaction to greatly increase the efficacy of CDT (Liu et al., 2019; Danewalia and Singh, 2021). Recently, Shen et al. (2020) reported MnFe_2_O_4_ NPs functionalized with Ir (III) complexes (Ir@MnFe_2_O_4_ NPs) for magnetic hyperthermia therapy (MHT) enhanced CDT. Cyclometalated iridium (III) complexes effectively targeted tumor cells inner mitochondrial membrane with high membrane potential. MnFe_2_O_4_ with superparamagnetism responded quickly to external magnetic fields and had excellent magnetothermal-properties including high magnetothermal conversion efficiency and magnetothermal stability. Therefore, Ir@MnFe_2_O_4_ NPs were internalized into cancer cells via the EPR effect and localized in mitochondria with help of cyclometalated iridium (III) complexes. GSH in cancer cells reduced the Fe (III) to Fe (II) on Ir@MnFe_2_O_4_ NPs surfaces to catalyze the Fenton reaction. More importantly, Ir@MnFe_2_O_4_ NPs generated high temperatures locally in the mitochondria under the alternating magnetic field (AMF), which produced a high level of ROS to induce mitochondria damage by greatly promoting the rate of the Fenton reaction. Ir@MnFe_2_O_4_NPs + AMF demonstrated a much stronger effect of inhibiting tumor growth than Ir@MnFe_2_O_4_NPs and AMF due to the synergistic effect of MHT and CDT.

Local high temperature through near-infrared light is also an effective strategy to enhance the efficacy of CDT (Zhou et al., 2019; Li et al., 2021b). For example, Jin et al. (2020) recently reported dual-targeted core-shell nanoplatforms (PTFHD nanoplatforms) for photothermal therapy (PTT) enhanced CDT. The PTFHD nanoplatforms were composed of hyaluronic acid (HA) and TPP co-modified polydopamine (PDA) nanoparticles loaded with DOX and Fe ions. HA and TPP mediated the sequential multistage-targeting of PTFHD nanoplatforms from cancer cells to their mitochondria. The PTFHD nanoplatforms catalyzed Fenton reaction by Fe ions in mitochondria after enzymolysis of HA shell and exposure of PDA-TPP-Fe core. PDA was a high-efficiency photothermal agent with high photothermal conversion efficiency. The PTFHD nanoplatforms lead to the local high temperature of mitochondria site under the irradiation of near-infrared laser (808 nm), which greatly promoted the rate of the Fenton reaction and damages the mitochondria. The second near-infrared (1,000–1,350 nm, NIR-II) has deeper tissue penetration and higher maximum allowable exposure than the first near-infrared (750–1,000 nm, NIR-I) light (Wang et al., 2021e; Liu et al., 2021; Zhang et al., 2021). Very recently, Li et al. (2021c) developed mitochondria-targeted MoS_2_@PDA-Fe@PEG/TPP nanosheets (MPFPT NSs) for the combination therapy of NIR-II-activated PTT and CDT. MPFPT NSs had a high photothermal conversion efficiency at NIR-II (34.9%). In addition, the Fe doped in MPFPT NSs was adopted as a Fenton reaction catalyst. MPFPT NSs significantly led to mitochondrial dysfunction and cancer cell apoptosis through TPP-mediated mitochondria targeting under NIR-II light (1,064 nm). The MPFPTNSs had a better tumor suppressive effect than similar nanoagents without mitochondria targeting ability or relying on single-mode therapy thanks to the superiority of PTT-CDT with mitochondria targeting.

Iron-based Fenton reaction has a low reaction rate. In addition, the optimal pH value for iron-based Fenton reaction is usually pH 2-5, and the reaction rate is low in the neutral intracellular environment of tumor cells (pH7.4) (Bao et al., 2021). The Fenton reaction rate of Cu(I) (1 × 10^4^ M^−1^s^−1^) is much higher than that of Fe (II) (76 M^−1^s^−1^) (Hu et al., 2019; Li et al., 2019; Lin et al., 2019). Meanwhile, the pH range required for the reaction of Cu(I) is wider than that of Fe(II), which can be carried out in weakly acidic and neutral media (Ma et al., 2019). Recently, Li et al. (2020) proposed an exo/endogenous dual-augmented mitochondria targeting nanoplatform (CuO@AuCu-TPP) for PTT-enhanced CDT by Cu(I) mediated Fenton reaction. The CuO@AuCu was prepared by *in-situ* reduction of chloroauric acid on Cu_2_O truncated octahedron. In this process, the Cu(I) in truncated octahedron was converted to Cu (II), and chloroauric acid was reduced to small gold nanoparticles on the surface of Cu_2_O truncated octahedrons. The CuO@AuCu were further modified with TPP (CuO@AuCu-TPP) to target the mitochondria of cancer cells. The aggregates of small gold nanoparticles on the surface of CuO@AuCu-TPP were adopted as a powerful photothermal agent because of their strong near-infrared absorption. Cu (II) was reduced into Cu (I) by GSH after CuO@AuCu-TPP were enriched in mitochondria, which deleted GSH and initiated a rapid Fenton reaction. Moreover, ROS generation efficiency of the CuO@AuCu-TPP further increased by 4.6 times under the irradiation of near-infrared light (808 nm). The CuO@AuCu-TPP possessed an excellent antitumor effect due to the PTT enhanced CDT and GSH depletion in Balb/c mice tumor models.

## Conclusion and Prospect

Traditional anticancer drugs including CDT agents are limited by their poor pharmacokinetic properties, such as low bioavailability, short half-life and low stability. Fortunately, nanotechnology can effectively improve the pharmacokinetic characteristics of traditional drugs by changing the size, shape and material characteristics of nanomaterials (Liu et al., 2012; Chen et al., 2021; Zhao et al., 2022; Zhu et al., 2022). Especially, well-designed nanodrugs can greatly increase the penetration of drugs at tumor sites and improve their pharmacokinetic and pharmacodynamic properties by the EPR effect. After decades of development, some nanodrugs have been approved for clinical cancer treatment, such as liposomes, polymer micelles, and inorganic nanomaterials (Cheng et al., 2021). However, most of these nanodrugs are carried with traditional chemotherapeutic drugs. Fundamentally, these nanodrugs are difficult to solve the resistance of cancer cells to traditional chemotherapeutic drugs, and have limited efficacy for cancer. Mitochondria are probably the most vulnerable part of cancer cells, because mitochondria not only play an irreplaceable core role in cancer cells but are also a key link in the apoptosis pathway of cancer cells. Therefore, mitochondria are likely to be the Achilles heel of cancer cells. Compared with non-mitochondria-targeting treatments, M-CDT nanodrugs exhibit higher therapeutic effect with lower dosage of drugs via precise and severe damage to mitochondria. More importantly, M-CDT nanodrugs are expected to solve the resistance of traditional drugs, because the resistance of cancer cells is generally ATP-dependent. For example, P-glycoproteins use ATP produced by mitochondria to pump a variety of anti-cancer drugs with different structures and different molecular weights out of the cancer cell, which is why cancer cells acquire multiple drug resistance. These emerging M-CDT nanodrugs destroy mitochondria very efficiently and fundamentally eliminate the multi-drug resistance of cancer cells. In short, M-CDT nanodrugs have great advantages over traditional drugs and conventional CDT nanodrugs, and have demonstrated amazing effects of cancer treatment. However, M-CDT nanodrugs still have some challenges to overcome.

First, M-CDT nanodrugs with the classic iron-based Fenton reaction face the bottleneck of insufficient reaction rate. Iron is a ubiquitous element in the human body and has excellent biocompatibility. However, the biggest problem lies in its low efficiency under the conditions of the cancer microenvironment. Hyperthermia can increase the reaction rate, but it brings complexity to the treatment and does not fundamentally solve the problem. In addition, other transition metals such as Cu, Pt, etc. are adopted to increase the reaction rate. However, the biocompatibility of transition metals is far less than that of iron (Ai et al., 2021). Reducing the pH of cancer cells is a very promising solution. The deduction of pH in tumor cells reinforces the response rate of the classic Fenton reaction-based CDT and simultaneously induces mitochondrial calcium overload, and these two resulting effects synergistically cause mitochondrial dysfunction and therefore tumor cell death (Bao et al., 2021). Secondly, for non-H_2_O_2_-dependent M-CDT nanodrugs, precise control of CDT drugs release on the mitochondria is a great challenge. Most of these CDT drugs can produce ROS only when they interfere with the mitochondrial respiratory chain in the inner mitochondrial membrane. In addition, these CDT drugs generally belong to traditional high toxic chemotherapy drugs, and their uncontrollable release can cause great side effects. Third, cancer cells themselves have sophisticated antioxidant defense systems, such as abundant GSH and antioxidant enzyme systems, etc (Su et al., 2021). Therefore, M-CDT nanodrugs often need to intervene in the various factor of antioxidant defense systems. Integrating multiple functions on M-CDT nanodrugs is still a great challenge.

Nevertheless, M-CDT nanodrugs are very promising in cancer treatment. M-CDT nanodrugs will be undergoing rapid development with a deeper understanding of tumor pathology and treatment methods. We hope that a new way for the clinical transformation of the M-CDT nanodrugs will be opened through the optimized design of M-CDT nanodrugs.
